# Chaperone-Assisted Mitotic Actin Remodeling by BAG3 and HSPB8 Involves the Deacetylase HDAC6 and Its Substrate Cortactin

**DOI:** 10.3390/ijms22010142

**Published:** 2020-12-25

**Authors:** Carole Luthold, Alice-Anaïs Varlet, Herman Lambert, François Bordeleau, Josée N. Lavoie

**Affiliations:** 1 Centre de Recherche sur le Cancer, Université Laval, Québec, QC G1R 3S3, Canada; carole.luthold@crchudequebec.ulaval.ca (C.L.); aav57@cornell.edu (A.-A.V.); hermanlambert007@gmail.com (H.L.); 2 Oncology, Centre de Recherche du CHU de Québec-Université Laval, Hôtel-Dieu de Québec, Québec, QC G1R 3S3, Canada; 3 Département de Biologie Moléculaire, Biochimie Médicale et Pathologie, Faculté de Médecine, Université Laval, Québec, QC G1V 0A6, Canada

**Keywords:** BAG3 1, HSPB8 2, p62/SQSTM1 3, HDAC6 4, Arp2/3 5, cortactin 6, mitosis 7, actin cloud 8, actin remodeling 9

## Abstract

The fidelity of actin dynamics relies on protein quality control, but the underlying molecular mechanisms are poorly defined. During mitosis, the cochaperone BCL2-associated athanogene 3 (BAG3) modulates cell rounding, cortex stability, spindle orientation, and chromosome segregation. Mitotic BAG3 shows enhanced interactions with its preferred chaperone partner HSPB8, the autophagic adaptor p62/SQSTM1, and HDAC6, a deacetylase with cytoskeletal substrates. Here, we show that depletion of BAG3, HSPB8, or p62/SQSTM1 can recapitulate the same inhibition of mitotic cell rounding. Moreover, depletion of either of these proteins also interfered with the dynamic of the subcortical actin cloud that contributes to spindle positioning. These phenotypes were corrected by drugs that limit the Arp2/3 complex or HDAC6 activity, arguing for a role for BAG3 in tuning branched actin network assembly. Mechanistically, we found that cortactin acetylation/deacetylation is mitotically regulated and is correlated with a reduced association of cortactin with HDAC6 in situ. Remarkably, BAG3 depletion hindered the mitotic decrease in cortactin–HDAC6 association. Furthermore, expression of an acetyl-mimic cortactin mutant in BAG3-depleted cells normalized mitotic cell rounding and the subcortical actin cloud organization. Together, these results reinforce a BAG3′s function for accurate mitotic actin remodeling, via tuning cortactin and HDAC6 spatial dynamics.

## 1. Introduction

Key biological processes rely on a strict control of cell shape change through remodeling of the actin cortex, including cell migration, differentiation, and mitotic cell division [1,2]. Mitotic cell division is undoubtedly the most dramatic cell shape change that relies on a reversible reorganization of the overall cellular structures, typically within minutes, thereby providing a paradigm to identify key regulatory mechanisms. Whether in tissue-culture or inside tissues, mitotic cell rounding is essential for faithful spindle orientation and chromosome capture [3,4,5]. Rapid disassembly of the interphase actin network and focal adhesions at mitotic entry enables the formation of a rigid actomyosin cortex that drives mitotic cell rounding. This process is facilitated by the targeted degradation of cortical proteins [6]. Timely mitotic cell rounding also crucially relies on a spatial control of distinct actin nucleation activities to assemble the contractile actin cortex. A switch from Arp2/3 complex-mediated branched actin network assembly to Diaphanous-mediated actin nucleation regulates the necessary changes in cortical actomyosin organization [7,8,9]. However, in rounded mitotic cells, Arp2/3 complex is required to generate a polarized and highly dynamic subcortical actin network (the actin cloud) that responds to forces and contributes to spindle orientation [10,11]. Thus, rapid changes in the actin network organization are needed for proper spindle positioning and precise chromosome segregation. How the fine-tuning of mitotic actin remodeling is achieved remains poorly understood.

Protein quality control (PQC) can resolve damage at the level of proteins, cytoskeletal structures, or organelles, and assist protein complex assembly to ensure their appropriate dynamics via cellular processes that promote the spatial sequestration of proteins, their recycling, or their degradation [12,13]. Recent work has supported the implication of PQC factors in the spatial regulation of actin remodeling processes relying on assembly and disassembly of higher-order macromolecular structures that are at risk of collecting damage. In early mitosis, vesicles containing the autophagic cargo receptor NDP52 can sequester the actin nucleation-promoting factor N-Wasp and limit actin filament growth at the cell cortex [14]. This mechanism facilitates the necessary changes in cortical actin organization for precise spindle positioning. At mitotic exit, another multi-functional autophagic receptor, p62/SQSTM1, targets Ras homolog family member A (RhoA) for autophagic degradation to limit actin filament assembly at the midbody [15]. Our recent finding of a dynamic between p62/SQSTM1, HSPB8 and the cochaperone BCL2-associated athanogene 3 (BAG3) further points to an important role for PQC factors in assisting accurate actin remodeling during mitotic cell division [16,17].

Molecular chaperones of the heat shock protein (HSP) family and their cochaperones play crucial roles in PQC [18,19]. BAG3 belongs to the BAG family of cochaperones that regulate the fate of HSP70 client proteins [20,21]. However, BAG3 uniquely functions as a molecular scaffold for recruiting chaperones of the small heat shock protein (sHSP/HSPB) family, which are linked to the stability and dynamics of the actin network [13,22,23,24,25]. BAG3 also interacts with autophagic factors and signaling proteins and thereby exerts multifaceted functions in cytoskeletal dynamics, nuclear envelope integrity, cell survival, and selective autophagy [20,26]. Recent work has suggested that BAG3-containing chaperone platforms mainly act by sequestering cellular activities and/or proteins destined for degradation during stress adaptive responses, as well as during physiological cytoskeletal remodeling [27,28,29,30]. In this regard, we recently uncovered a function for BAG3 in accurate mitotic progression that implicates HSPB8 and p62/SQSTM1 [16]. BAG3, as a typical mitotic regulator, is hyper-phosphorylated at mitotic entry and localizes to the nuclear envelope and centrosomal regions. BAG3 depletion hinders mitotic cell shape remodeling, causing multiple defects in mitotic cell rounding, actin retraction fibers and spindle positioning. This BAG3 function is highly correlated with phenotypes that are typically caused by faulty remodeling of the mitotic actin network [16]. Moreover, depletion of BAG3 or HSPB8 also causes actin-related defects in cytokinesis that can be mimicked by inhibitors of lysosomal function, suggesting a role in cytoskeletal PQC [17]. However, the mechanisms underlying BAG3′s mitotic function remain to be defined. Intriguingly, the BAG3 chaperone complex recruits histone deacetylase 6 (HDAC6) in early mitosis, which have known cytoskeletal substrates [16,31].

In this context, we hypothesized that a BAG3 mitotic function implicating HDAC6 contributes to maintaining the fidelity of actin dynamics during mitosis. In this study, we show that the depletion of BAG3 and its mitotic partners, HSPB8 and p62/SQSTM1, recapitulated the same mitotic cell rounding defects that were normalized by the downregulation of Arp2/3 complex and HDAC6 activity. Furthermore, we uncovered a regulatory role for these PQC factors in the dynamic of the subcortical actin cloud, a poorly characterized actin network in round mitotic cells that plays a role in spindle positioning [10,11]. The results suggest that BAG3 acts to spatially restrict Arp2/3 complex during mitosis, by controlling interactions between HDAC6 and its substrates, notably the actin nucleation-promoting factor cortactin. This work adds to the evidence that a cytoskeletal PQC mechanism involving BAG3 and HSPB8 is needed to ensure faithful actin remodeling during crucial cell shape transitions. Understanding the biology of BAG3′s mitotic function should be relevant for the targeting of cancer cell plasticity and invasive behavior.

## 2. Results

### 2.1. Downregulation of HDAC6 Activity Can Normalize Mitotic Cell Rounding in Cells Depleted of BAG3, HSPB8 or p62/SQSTM1

**Figure 1:**
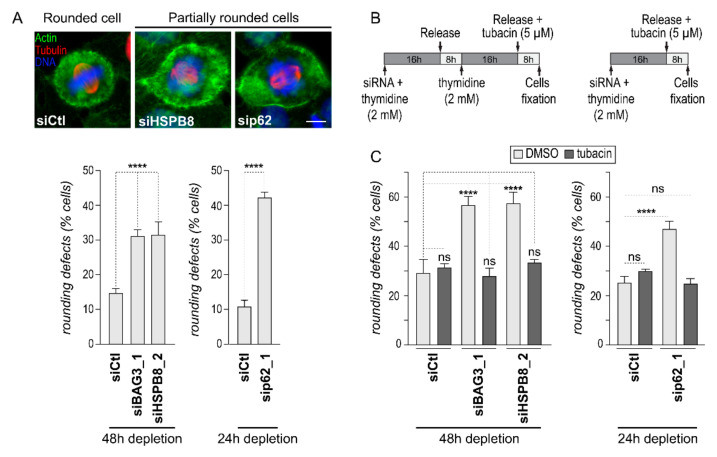
Downregulation of HDAC6 activity can normalize mitotic cell rounding in cells depleted of BAG3, HSPB8 or p63/SQSTM1. (**A**) Epifluorescence images of HeLa-H2B-RFP cells showing a typical mitotic morphology (siCtl) as compared to cell rounding defects (sip62, siHSPB8). The graph shows quantification of the percentages of mitotic cells treated with the indicated siRNAs that displayed rounding defects (flat or partially rounded cells); means ± SEM (*n* = 609 to 817 cells from *N* = 3 experiments). Scale bar: 10 μm. (**B**) Schematics of the protocols; phenotypes were quantified on fixed cells 24 h or 48 h after siRNA transfection, as indicated. (**C**) The graphs depict mitotic cell rounding defects (flat or partially rounded cells); means ± SEM (*n* = 591 to 633 cells from 3 experiments). Statistical analyses were performed by the chi-square test; ****, *p* < 0.0001; ns, non-significative.

We have previously shown that BAG3 forms a mitotic complex with HSPB8, p62/SQSTM1, and HDAC6 [16]. To define the role of this chaperone complex in mitotic actin remodeling, we first assessed the impact of each of these protein partners on mitotic cell rounding, using HeLa cells that have widely characterized mitotic phenotypes. HeLa cells expressing a fluorescent histone marker (HeLa-H2B-RFP) were transfected with small interfering RNA(siRNA) sequences targeting either BAG3, HSPB8, or p62/SQSTM1, and were synchronized in mitosis (Figure 1 and Figure S1A–C). Mitotic cell rounding was visualized by staining of F-actin and α-tubulin in fixed cells. In agreement with our previous finding, BAG3 depletion increased by ~2-fold the proportion of cells showing rounding defects as compared to control cells (Figure 1A; partially round or flat mitotic cells; compare cells treated with BAG3-specific siRNAs [siBAG3] to cells treated with control siRNA [siCtl]) [16]. Importantly, depletion of HSPB8 or p62/SQSTM1 recapitulated the BAG3 phenotype on mitotic cell rounding (Figure 1A). Two different siRNA sequences for each protein gave essentially the same inhibition of mitotic rounding (Figure S1B). Of note, the effect of p62/SQSTM1 depletion was analyzed at an earlier depletion time (24 h) due to a decrease in mitotic cell integrity at 48 h. Thus, the results suggest that BAG3 acts together with its mitotic partners in PQC to facilitate timely mitotic cell rounding.

We then sought to determine the role of HDAC6 in this process. Since a 48 h treatment with HDAC6-specific siRNA interfered with mitotic cell rounding (Figure S1C), we used a more targeted pharmacological treatment with the HDAC6 inhibitor tubacin to limit our analysis to mitotic events [32]. Cells were subjected to an 8 h treatment with tubacin following cell synchronization in S phase (Figure 1B). Under these conditions, tubacin-treated control cells did not present significant cell rounding defects (Figure 1C). In marked contrast, tubacin brought back the proportion of mitotic cell rounding defects to the level observed in control cells in cells depleted of BAG3, HSPB8, or p62/SQSTM1 (Figure 1C). Western blot analysis of the levels of acetylated tubulin, a major substrate of HDAC6, confirmed the efficiency of tubacin treatment in these cells (Figure S1D) [32,33]. Since HDAC6 inhibition can normalize mitotic cell rounding in the absence of a functional BAG3 mitotic complex, BAG3 mitotic complex may act by limiting HDAC6 activity.

### 2.2. BAG3, HSPB8, and p62/SQSTM1 Are Required for Faithful Subcortical Actin Cloud Dynamic in Round Mitotic Cells

**Figure 2:**
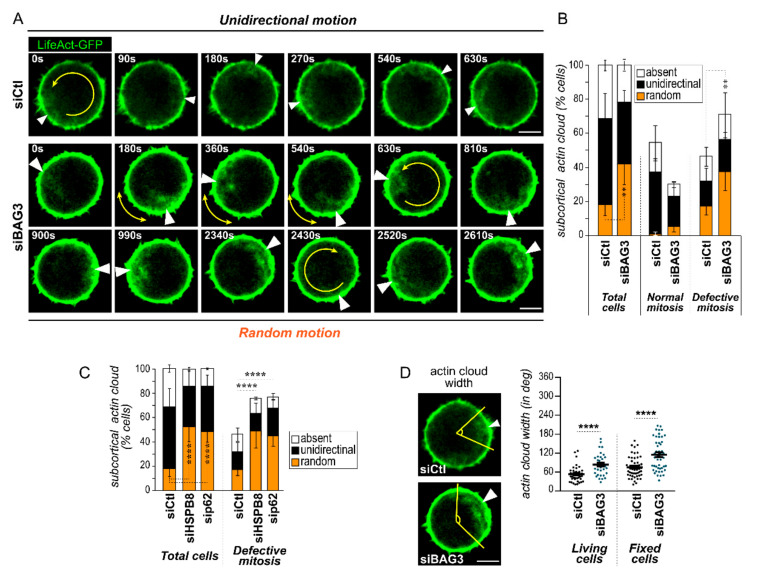
BAG3 and its mitotic partners regulate the dynamic and organization of the mitotic actin cloud. (**A**) Video stills taken from live-cell imaging of HeLa-H2B-RFP cells infected with LifeAct-GFP, showing representative dynamics of the subcortical actin cloud in control cells (siCtl; sustained and unidirectional motion), as compared to cells transfected with BAG3-specific siRNA (siBAG3; random motion); the white arrow points to the subcortical actin cloud and the yellow arrows indicate the direction of the revolving movement. See also Videos S1 and S2. (**B**) Quantification of the revolving movement behaviors for datasets represented in (**A**) within mitotic cell populations (total cells; **left graph**), within subpopulations of cells with normal mitotic phenotypes, or within subpopulations of cells showing mitotic defects (defective mitosis: spindle rocking and delay in prometa-and metaphases; **right graph** side); means ± SEM (*n* = 97 to 139 cells from *N* = 3 experiments). (**C**) Quantification of the revolving movement behaviors from time lapse analyses of HeLa-H2B-RFP expressing LifeAct-GFP and transfected with control siRNA (siCtl) or HSPB8- (siHSPB8) or p62/SQSTM1-specific siRNAs (sip62) within the indicated mitotic cell populations; please note that the control cell populations datasets are the same as in B. The data are the means ± SEM (*n* = 97 to 139 cells from *N* = 3 experiments). Statistical analyses were performed using the chi-square test; **, *p* < 0.01; ****, *p* < 0.0001. (**D**) Representative confocal images showing the organization of the actin cloud; the angular widths are delineated by yellow lines and positions of the actin clouds are pointed out by white arrowheads. Scale bar: 10 µm. The graph shows the actin cloud angular widths in live cells expressing LifeAct-GFP (**left**) or in fixed cells stained with fluorescent phalloidin (**right**), as measured using the method described in Figure S2; means +/− SEM of *n* = 35 and 53 cells from 3 experiments. Statistical analysis was performed using the Mann–Whitney test; ****, *p* < 0.0001.

Accurate spindle positioning in round mitotic cells relies on a highly dynamic subcortical actin structure [10,11]. Typically, this actin cloud performs a circular motion along the cell cortex from prometaphase to anaphase. Considering that a large proportion of BAG3-depleted cells underwent mitotic rounding (Figure 1A), we investigated whether BAG3 depletion could also interfere with the subcortical actin cloud dynamic. To do so, we monitored subcortical actin cloud spatiotemporal dynamics by live-cell imaging using LifeAct-GFP to label actin structures. The majority of control cells displayed a dynamic F-actin agglomerate located close to the mitotic cortex that was reminiscent of the previously described mitotic actin cloud (Figure 2A,B, siCtl). Further categorization of the actin cloud dynamics in control cells revealed that the rotation movement was mostly sustained and unidirectional (~50%; Video S1), whereas it occasionally displayed random and sudden changes in direction (~18%) (Figure 2B, siCtl). Notably, most of the cells that displayed a random motion of the actin cloud also showed defects in spindle positioning and/or mitotic progression (Figure 2B; siCtl, orange bars). Remarkably, BAG3 depletion significantly increased the proportion of mitotic cells showing random actin cloud motion (~42%; Figure 2A,B, siBAG3; Video S2). This increase in random actin cloud dynamics correlated with higher levels of spindle positioning and mitotic progression defects in BAG3-depleted cells (Figure 2B, “defective mitosis”). Depletion of HSPB8 or p62/SQSTM1 similarly interfered with the actin cloud dynamic (Figure 2C). These results suggest that BAG3 and its mitotic partners facilitate fine-tuning of the subcortical actin structure dynamic.

We then sought to analyze BAG3′s effect on actin cloud organization by monitoring the spatial distribution of subcortical actin structures in both live and fixed cells. To do so, we measured the subcortical actin cloud angular distribution from images of actin-labeled cells displayed in radial coordinates (Figure S2). In LifeAct-GFP-expressing live cells, the average angular width of the actin cloud was 66.6° ± 6.73 in control cells, while it was 88.05° ± 5.99 in BAG3-depleted cells (~1.3-fold wider, Figure 2D). Similarly, we found that BAG3 depletion increased the angular width of the actin cloud by ~1.75-fold in fixed cells (156° ± 13.4 in siBAG3 cells versus 89°± 5.9 in control cells; Figure 2D). These results suggest that BAG3 contributes to faithful spindle orientation, by facilitating the spatiotemporal remodeling of the subcortical actin cloud.

### 2.3. Downregulation of Arp2/3 Complex or HDAC6 Activity Normalizes Actin Cloud Organization and Dynamic in BAG3-Depleted Cells

**Figure 3:**
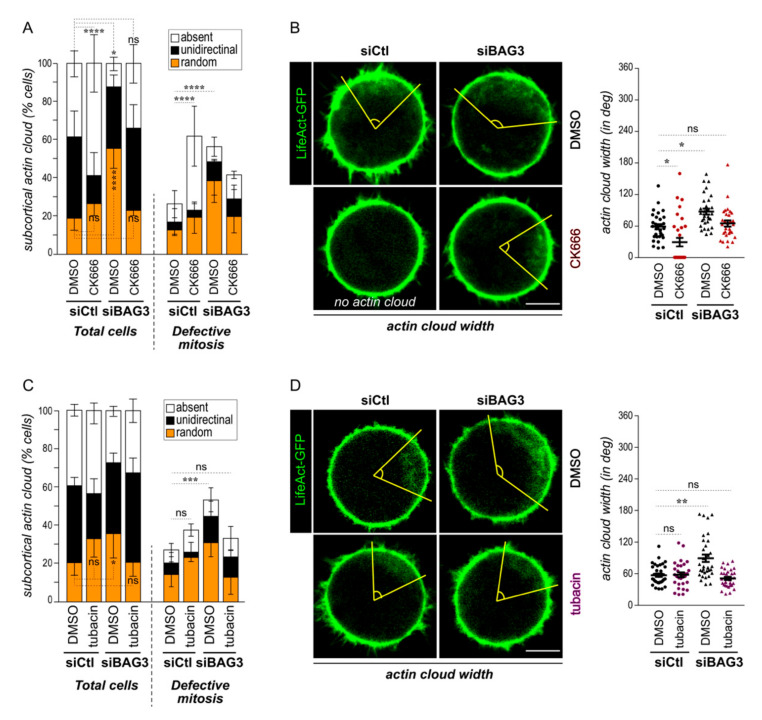
Downregulation of Arp2/3 or HDAC6 activity in BAG3-depleted cells can normalize the actin cloud dynamic. (**A**,**C**) Quantification of the actin cloud motion from time lapse analyses of HeLa-H2B-RFP cells that have been transfected with the indicated siRNAs and incubated with Arp2/3 inhibitor CK666 (40 µM; **A**) or HDAC6 inhibitor tubacin (20 nM; **C**), in total cell populations as compared to cells showing mitotic defects (spindle rocking or delayed in prometaphase); means ± SEM of *n* = 69 and 146 cells from 2 to 3 experiments. (**B**,**D**) Representative confocal images showing the organization of the actin cloud; the angular widths are delineated by yellow lines. Scale bars: 10 µm. Graphs show the actin cloud angular widths in live cells expressing LifeAct-GFP (**left**) or in fixed cells stained with fluorescent phalloidin; data are the means ± SEM of *n* = 30–34 cells from 2 to 3 independent experiments. Statistical analyses were performed using the Mann–Whitney test; *, *p* < 0.05; **, *p* < 0.01, ***, *p* < 0.001; ****, *p* < 0.0001.

Assembly and the dynamic of the mitotic subcortical actin cloud depend on the Arp2/3 complex [10]. Actin polymerization and depolymerization events are required to generate and restrict the actin cloud circular motion to a “moving zone”. We reasoned that dysregulation of the angular width of the actin cloud in BAG3-depleted cells could be due to a lack of Arp2/3 complex control. To test this, we first analyzed the impact of Arp2/3 inhibition on the actin cloud dynamic in BAG3-depleted cells using the Arp2/3 complex inhibitor CK666. Live-cell imaging of LifeAct-GFP-expressing cells revealed that inhibition of Arp2/3 activity increased the proportion of control cells that lack a subcortical actin cloud (~1.5-fold; Figure 3A, siCtl). In line with this result, the angular width of the subcortical actin cloud was markedly decreased in the remaining cells that still presented subcortical actin clusters (38° ± 11.3 in CK666-treated cells versus 71° ± 9.3 in DMSO-treated cells, Figure 3B). Remarkably, the actin cloud dynamic was normalized following CK666 treatment of BAG3-depleted cells, as revealed by the similar behavior of the actin cloud motion compared to the control cells (Figure 3A, siBAG3). Moreover, the CK666 treatment in these cells brought the angular width of the actin cloud to near the control cell levels (65° ± 5.7; Figure 3B). Additionally, the CK666 treatment reduced the proportion of BAG3-depleted cells with spindle positioning defects and mitotic delays, whereas the same treatment increased these mitotic defects in control cells (Figure 3A; defective mitosis). Finally, a pilot experiment revealed that the CK666 could also decrease cell rounding defects in BAG3-depleted cells (Figure S3). Under these conditions, CK666 had little impact on mitotic cell rounding in control cells. Together, these results suggest that BAG3 may act, in part, by restricting Arp2/3 activity.

We next asked whether HDAC6 inhibition, which normalized mitotic cell rounding in BAG3-depleted cells (Figure 1C), could also correct the actin cloud dynamic. To limit the effect of HDAC6 inhibition, tubacin was added 30 min before performing time lapse imaging of HeLa cells expressing LifeAct-GFP and α-tubulin-RFP. We observed that high doses of tubacin (5 µM) led to an almost complete disappearance of the subcortical actin cloud and also impaired spindle positioning in control cells (Figure S4). In this context, we selected an optimized dose of tubacin (20 nM) that did not significantly interfere with these events in control cells (Figure 3C). Under these conditions, the proportion of BAG3-depleted cells showing a random actin cloud dynamic associated with mitotic spindle mispositioning and/or mitotic delay was brought back to near the control levels (Figure 3C). Additionally, the angular width of the actin cloud was normalized in tubacin-treated BAG3-depleted cells (Figure 3D). These results indicate that the generation and dynamic of the mitotic actin cloud is highly sensitive to HDAC6 inhibition, suggesting that HDAC6 is a novel regulator of this mitotic-specific actin structure. Furthermore, they also suggest that BAG3 acts by limiting HDAC6 activity towards relevant substrate controlling the actin cloud dynamic.

### 2.4. BAG3 Limits the Mitotic Association of HDAC6 with Its Substrate Cortactin, an Arp2/3 Regulatory Protein that Modulates Mitotic Actin Remodeling

HDAC6 can modulate branched actin polymerization by deacetylating cortactin [34,35,36]. Deacetylation of cortactin enhances its ability to promote Arp2/3-mediated F-actin branched polymerization. We reasoned that the BAG3–HSPB8–p62/SQSTM1 mitotic complex, which recruits HDAC6 [16], could affect Arp2/3-dependent functions by modulating HDAC6–cortactin interaction.

**Figure 4:**
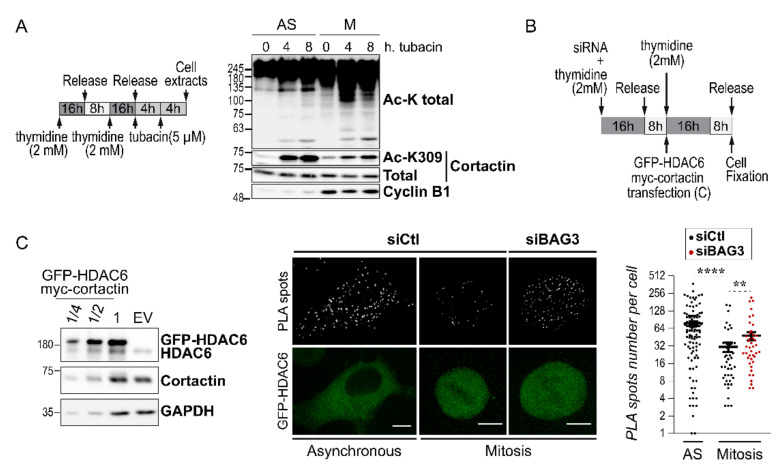
BAG3 depletion impairs the mitotic regulation of HDAC6–cortactin association. (**A**) Schematic of the protocol used to analyze protein acetylation. Representative Western blots showing the acetylation levels of total proteins (Ac-K) or cortactin acetylation (Ac-K309) in asynchronous (AS) versus mitotic HeLa cells (M); cyclin B1 levels were monitored as a marker of mitosis; total cortactin: loading control. (**B**) Schematic of the protocol used to assess HDAC6–cortactin association in situ. (**C**) Representative Western blots showing GFP-HDAC6 and myc-cortactin levels after transfection in HeLa cells; Glyceraldehyde 3-phosphate dehydrogenase (GAPDH): loading control, EV: empty vector. Representative confocal images show proximity ligation assay (PLA) signal using anti-GFP and anti-cortactin antibodies, and GFP-HDAC6 in an asynchronous cell versus a mitotic cell transfected with control siRNA (siCtl), as compared to a mitotic cell depleted of BAG3 (siBAG3). Scale bars: 10 µm. The graph shows quantification of the number of PLA foci per cell; means +/− SEM (*n* ≥ 30 cells; *N* = 3 experiments). Statistical analysis was performed by the Mann–Whitney test; ****, *p* < 0.0001; **, *p* < 0.01.

As a first step, we measured cortactin acetylation changes in mitotic cells as compared to asynchronous cells. Asynchronous or synchronized mitotic cells were treated with 5 µM of tubacin for either 4 h or 8 h (Figure 4A, experimental scheme). Western blot analyses of total acetylation levels and cortactin acetylation levels were then performed using a broad-spectrum anti-acetyl antibody (Ac-K total), and an antibody against cortactin acetylated lysine 309 that is targeted by HDAC6 (Ac-K309 cortactin) (Figure 4A) [34]. As expected, total level of protein acetylation increased in tubacin-treated cells in both asynchronous and mitotic cells, showing that HDAC6 is active in mitotic cells (Figure 4A, total Ac-K). However, we noted that mitotic cells exhibited a distinct protein acetylation pattern. Notably, inhibition of HDAC6 with tubacin was associated with lower increases in acetyl-cortactin levels in mitotic cells relative to those observed in asynchronous cells (Figure 4A, Ac- K309, compare M versus AS).

We then sought to assess the impact of BAG3 on cortactin acetylation during mitosis. To do so, we used an indirect assay to measure single-cell cortactin–HDAC6 interaction in situ, as a biochemical analysis was not sensitive enough to measure variations in cortactin acetylation using a knockdown approach in heterogenous cell populations. We therefore exploited a Duolink^®^ proximity ligation assay (PLA) to visualize protein–protein interactions in situ [37]. HeLa cells treated with siRNA were cotransfected with GFP-HDAC6 and myc-cortactin, synchronized in mitosis, and then processed for PLA detection (Figure 4B,C). Numerous fluorescent foci were detected in asynchronous control cells (~74 PLA foci/cell; Figure 4C, siCtl, asynchronous). However, foci numbers were significantly decreased in siCtl-mitotic cells, indicating that HDAC6–cortactin association is downregulated in mitosis (~30 foci/cell; Figure 4C, siCtl, mitosis). Strikingly, BAG3 depletion increased by nearly 2-fold the number of PLA foci in mitotic cells compared to control cells, thereby preventing the mitotic decrease in HDAC6–cortactin association (Figure 4C, siBAG3).

**Figure 5:**
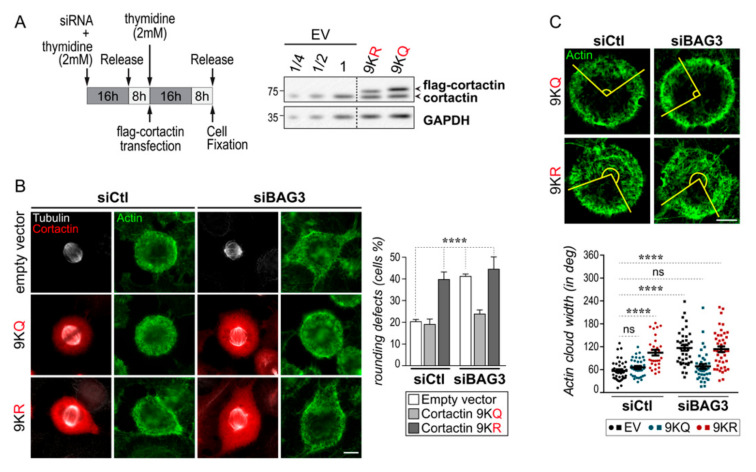
Acetyl-mimic cortactin-9KQ can normalize mitotic cell rounding and actin cloud organization in BAG3-depleted cells. (**A**) Schematic of the protocol. Representative Western blots showing the expression of cortactin-9KR and cortactin-9KQ mutant after transfection in HeLa cells; GAPDH: loading control. EV: empty vector. (**B**) Representative deconvolved epifluorescence images showing the mitotic rounding phenotypes induced by cortactin mutants. The graph depicts the percentages of cells with defects in mitotic cell rounding; means ± SEM of at least 609 cells from 3 experiments. Statistical analysis was performed by the chi-square test ****, *p* < 0.0001. (**C**) Representative confocal images showing the organization of the actin cloud; the angular widths are delineated by yellow lines. Scale bar: 10 µm. The graph shows the actin cloud angular widths in fixed cells stained with fluorescent phalloidin; data are the means ± SEM of n ≥ 33 cells; *N* = 3 experiments. Statistical analysis was performed using by the Kruskal–Wallis test and ****, *p* < 0.0001.

So far, the results suggest that BAG3, by targeting HDAC6, could interfere with cortactin deacetylation to limit Arp2/3 activity. To further explore this hypothesis, we took advantage of formerly characterized cortactin mutants that have been engineered to either disrupt (cortactin-9KR) or mimic acetylation (cortactin-9KQ) (Figure 5A) [34]. We found that expression of the acetyl-mimic cortactin-9KQ mutant did not interfere with mitotic cell rounding in control cells (Figure 5B, siCtl + 9KQ). In marked contrast, the acetyl-mimic cortactin mutant corrected the mitotic rounding defects in BAG3-depleted cells, as well as in HSPB8-depleted cells (Figure 5B, siBAG3 + 9KQ; Figure S4B, siHSPB8 + 9KQ). Moreover, BAG3-depleted cells expressing the acetyl-mimic cortactin displayed a normalized angular width of the actin cloud (Figure 5C, siBAG3 + 9KQ). Conversely, the non-acetylable cortactin-9KR mutant caused substantial rounding defects in control cells and increased the angular width of the actin cloud in these cells, thereby mirroring the effects of BAG3 depletion (Figure 5B,C, siCtl + 9KR). However, there was no further increase in the cell rounding defect or angular width of the actin cloud in BAG3-depleted cells expressing the non-acetylable cortactin-9KR mutant (Figure 5B,C, siBAG3 + 9KR).

Altogether, our data suggest that the mitotic chaperone complex organized by BAG3 contributes to faithful cell rounding and spindle positioning, by fine-tuning the HDAC6–cortactin interaction, thereby limiting Arp2/3 complex activation.

## 3. Discussion

This work identifies a function for BAG3 and its mitotic partners: HSPB8, p62/SQSTM1 and HDAC6, in the restriction of Arp2/3-mediated actin assembly during mitosis, which assist in accurate cell rounding and spindle positioning. Moreover, it describes a novel mitotic BAG3 phenotype that is typified by the spatial disorganization of a pool of cytoplasmic actin, the actin cloud, which is also normalized by the downregulation of Arp2/3 or HDAC6 activity. This function may involve the fine-tuning of cortactin deacetylation, as the state of cortactin acetylation can influence the mitotic BAG3 phenotypes. Since the BAG3–HSPB8 complex also limits Arp2/3-dependent actin accumulation at the intercellular bridge during cytokinesis, fine-tuning of branched actin polymerization might be a main function of this chaperone complex, which assists the rapid switches in actin organization during cell shape changes [17].

Whether this BAG3 function is part of a mitotic mechanism for the regulated sequestration of cytoskeletal components merits further investigation. The molecular assembly of p62/SQSTM1 oligomers into higher ordered inclusion bodies is regulated by BAG3 and HSPB8 [28]. These inclusion bodies provide a platform for protein sequestration, autophagosome assembly and segregation of signaling intermediates [28,29,38,39,40,41,42,43,44,45]. There is precedent for a role for BAG3-associated multichaperone complex in the spatial sequestration of signaling intermediates at actin fiber sites [29,30]. This process is shown to promote the local clearance of damaged actin-binding protein in muscle cells. However, whether autophagy is active or stalls in early mitosis is controversial [46]. Nonetheless, segregation of actin cortex components to p62-inclusion bodies in early mitosis could facilitate disassembly of the interphase actin network to facilitate mitotic cell rounding when proteostasis is limited. Such a mechanism could provide mitotic sorting stations for superfluous proteins destined for degradation or recycling. Spatial sequestration into p62-inclusion bodies could also facilitate the activation/deactivation of signaling complexes implicating HDAC6. It is relevant to note that cyclin A2-containing inclusions have been described in late mitosis, which assist in the spatial activation of RhoA [47,48]. While the detailed molecular mechanism remains to be defined, it is interesting to speculate on how this BAG3 function may contribute to the timed changes in mitotic cytoskeletal organization we have observed here.

There are many potential ways by which a BAG3 function in fine-tuning branched actin polymerization could facilitate mitotic actin remodeling. One is by the fine-tuning of centrosomal actin nucleation, a process that is regulated by cortactin and the Arp2/3 complex. Cortactin mediates the attachment of centrosomes to F-actin at the G2/M transition, which contributes to the driving force for centrosome separation [49]. Later, a transient increase in centrosomal actin nucleation is reported to downregulate microtubule nucleation as the cell exits mitosis [50]. Accordingly, timed activation/deactivation of the cortactin–Arp2/3 axis at the centrosome could coordinate various steps of mitotic actin remodeling with microtubule dynamics. Based on the spatial dynamic of BAG3 during mitosis, it is tempting to speculate that a mechanism involving the BAG3 chaperone complex and its effect on the HDAC6–cortactin signaling axis plays a role in these events. Indeed, BAG3 and HSPB8 are recruited to centrosomes at the G2/M transition and remain enriched at the spindle poles during M-phase [16]. In principle, they could influence centrosomal Arp2/3-dependent actin nucleation, and thereby microtubule nucleation. While no major changes in mitotic microtubule organization were so far observed in BAG3-depleted cells, we cannot exclude some effect on astral microtubule dynamics contributing to spindle instability.

Another possible way by which the BAG3–HDAC6–cortactin axis could act is by the downregulation of cortactin deacetylation and Arp2/3 activity at the cell cortex in early mitosis. Indeed, our results are consistent with a mechanism whereby BAG3 and cortactin acetylation would act on the same pathway to facilitate mitotic cell rounding. In agreement with this, mitotic cell rounding is normalized in BAG3-depleted cells by overexpressing an acetyl-mimic cortactin mutant (9KQ). Conversely, the non-acetylable cortactin mutant (9KR) induces similar levels of cell rounding defects in control cells, without increasing these levels further in BAG3-depleted cells. Our analysis of HDAC6-regulated protein acetylation also supports a reduction in cortactin deacetylation and/or an increase in acetylation in early mitosis. Such a mechanism could facilitate the switch to Diaphanous-mediated cortical actin nucleation that is required to initiate cell rounding [8]. There is evidence that rapid fine-tuning of the cortex structure relies on a mechanism to adjust the relative contribution of Diaphanous and Arp2/3 that are responsible of the bulk actin cortex [7]. However, to our knowledge, a role for cortactin acetylation/deacetylation in mitotic actin cortex remodeling has so far not been described. Since HDAC6 has other cytoskeletal targets, it would not be surprising that the BAG3 chaperone complex could also regulate other effectors of mitotic cell rounding, for instance, myosin heavy chain 9 (MYH9). In this regard, it is important to note that BAG3 can stabilize skeletal myosin heavy chain in muscle cells [51]. Contrary to cortactin, MYH9 acetylation promotes its binding to actin and actomyosin contractility [52]. By restricting MYH9 deacetylation, the BAG3 chaperone complex could assist in achieving the contractile activity required for mitotic cell rounding.

Finally, our finding that the mitotic BAG3 chaperone complex regulates the subcortical actin cloud dynamic is most intriguing. Previous work has identified the Arp2/3 complex as a crucial factor in the formation and revolving movement of this mitotic actin cluster [10]. In agreement with this, we observed that Arp2/3 complex inhibition specifically impairs actin cloud formation in control cells without affecting mitotic cell rounding. Conversely, downregulation of Arp2/3 in BAG3-depleted cells could normalize actin cloud organization and revolving movement, as well as spindle positioning defects. These results corroborate previous findings that the actin cloud dynamic relies on continuous actin polymerization and depolymerization [10]. They also suggest that failure to properly orient the spindle in BAG3-depleted cells is mostly due to faulty actin cloud dynamic due to impaired actin filament turnover. Remarkably, we found that actin cloud formation and dynamic are also exquisitely reliant on proper HDAC6 activity. It could be that the BAG3 chaperone complex provides a platform to restrict cortactin targeting by HDAC6, thereby facilitating actin turnover within the cloud. Nonetheless, we cannot exclude the contribution of additional mechanisms. We have shown previously that depletion of either BAG3, HSPB8 or p62/SQSTM1 impairs actin retraction fiber number and organization [16]. Since the plan of the actin cloud revolving movement is determined by actin retraction fibers, the BAG3 chaperone complex may also impact the actin cloud dynamic by fine-tuning these cell–substratum adhesions [10,11]. It is important to note, however, that actin retraction fibers do not rely on the Arp2/3 complex [10]. Thus, the BAG3 chaperone complex may act at multiple levels to facilitate the generation and dynamic of the subcortical actin cloud and accurate spindle orientation.

## 4. Materials and Methods

### 4.1. Cell Culture, Synchronization, siRNA Transfection and Cell Treatments

HeLa-RFP-H2B (gift from Sabine Elowe, Centre CRCHU de Québec-Université Laval) and parental HeLa cells were maintained in α-minimal essential medium (α-MEM) supplemented with 10% fetal bovine serum and grown in a humidified atmosphere with 5% CO_2_ at 37 °C. Cells were synchronized by a double thymidine block and were released for 8 h to 10 h in fresh medium, as described previously [16,53]. For knockdown experiments, cells were transfected overnight with 50 nM siRNA duplexes in calcium-phosphate transfection buffer (125 mM MgCl_2_, 140 mM NaCl, 25 mM HEPES (4-(2-hydroxyethyl)-1-piperazineethanesulfonic acid), 0.75 mM Na_2_HPO_4_) or using RNAimax (Invitrogen) following the manufacturer’s recommendations for HeLa-RFP-H2B and parental HeLa cells, respectively. Analyses were performed 24 h to 48 h later by Western blot, live cell imaging or immunofluorescence. The siRNA duplexes were based on human sequences and were purchased from Qiagen (HPP grade siRNA) or Thermo Fisher Scientific (standard A4 grade). Sequences of the sense strands are as follows:

siBAG3_1: 5′-CGAAGAGTATTTGACCAAA-3′;

siBAG3_2: 5′-GCAAAGAGGTGGATTCTAA-3′;

siBAG3_3: 5′-GATGTGTGCTTTAGGGAAT-3;

siHSPB8_1: 5′-CAGATAGGCTAGTGGTATT-3′;

siHSPB8_2: 5′-GCAGTGAATGCAAGGGTTATT-3′;

sip62_1: 5′-GGAAATGGGTCCACCAGGATT 3’;

sip62_2: 5′- AGACCAAGAACTATGACAT -3′;

si AllStars negative control, target sequence: 5′-CAGGGTATCGACGATTACAAA-3′.

To rescue mitotic cell rounding and analyze protein acetylation by Western blot, CK666 (40 µM) or tubacin (5 µM) was added to cells that had been synchronized in mitosis by a thymidine block, for a period of 4 to 8 h during the second release period. To rescue the dynamic and organization of the subcortical actin cloud, CK666 (40 µM) or tubacin (20 nM) was added to cells synchronized in mitosis 30 min prior to time lapse imaging and during all the imaging periods.

### 4.2. Adenovirus, Infection, Vectors and Transfection

To follow the dynamics of actin and tubulin during live cell imaging, cells were seeded on fibronectin-coated glass Petri dishes (MatTek Corp.) and synchronized by the double thymidine block method. The infection was carried out in α-MEM without Desoxyribonucleosides/Ribonucleosides supplemented with 10% FBS (α-MEM-minus) during the first thymidine block using the adenovirus Ad-LifeAct-TagGFP2 (60121, IBIDI) at a multiplicity of infection (MOI) of 1 pfu/cell and the baculovirus Bac-RFP-αtubuline (C10614) at 4 pfu/cell diluted in the BacMam 2.0 reagent. The siRNA directed against BAG3 was transfected during the same period in a calcium-phosphate transfection buffer, as described previously [53] Sixteen hours after adenofection, cells were washed three times with HEPES and released into fresh medium for 8 h before adding 2 mM of thymidine for the second thymidine block.

For PLA experiments, peGFP.N1-HDAC6 WT (Addgene # 36188) and myc-cortactin plamids (a kind gift from Dr Zhan, Department of Experimental Pathology, Jerome H. Holland Laboratory for the Biomedical Sciences, American Red Cross, Rockville, MD 20855, USA) were transfected in parental HeLa cells during the second thymidine block period using Lipofectamine 2000 (Invitrogen), following the manufacturer’s instructions. Sixteen hours after transfection, cells were washed with Phosphate-buffered saline (PBS: 137 mM NaCl, 2.7 mM KCl, 4.3 mM Na_2_HPO_4_, 1.47 mM KH_2_PO_4_) and released in fresh medium for 8 h to 9 h before cell fixation.

For rescue experiments with cortactin mutants, 3XFLAG-cortactin mutants (9KQ and 9KR; kind gift of Dr Seto, Washington University School of Medicine and Health Sciences, Washington, DC 20037, USA) were transfected in HeLa-H2B-RFP cells during the first released in mitosis after thymidine block using Lipofectamine 2000 (Invitrogen). Eight hours after transfection, cells were processed for the second thymidine block and fixed 8–9 h after being released in mitosis.

### 4.3. Antibodies and Chemicals

The following antibodies were used for Western blot, immunofluorescence and PLA analyses: rabbit anti-BAG3 LP11 antibody (1:10000) was developed against a complete recombinant human BAG3 fused with glutathione S transferase (LP11; 1:10000 ) [16]; rabbit anti-HSPB8 against a C-terminal peptide (NELPQDSQEVTCT; 1:1000) [22]; anti-p62 SQSTM1 (sc-28359; 1:200) and anti-GFP (sc-9996, 1:1000; Santa Cruz); anti-GFP (632593, 1:500; Clontech); anti-HDAC6 (#7612; 1:1000) and anti-lysine-acetylated (ac-K-100, #9814, 1:1000; Cell Signaling Technologies); anti-vinculin (V9131; 1:5000), anti-Flag M2 (F3165, 1/200) and anti-α-tubulin mouse (T5168, 1:10000; Sigma-Aldrich); anti-cortactin (ab33333, 1:1000) and anti-α-tubulin rabbit (ab18251, 1:1000; Abcam); anti-acetyl-cortactin K309 (ABT1378, 1:1000); anti-cyclin B1 (ac-245, 1:1000) and anti-GAPDH Clone 6C5 (Fitzgerald, #10R-G109a, 1:10000) were from Millipore.

The Alexa-Fluor^®^ 488 Phalloidin (A12379, 1/50) comes from Molecular Probes/Thermo Fisher. Tubacin (S2239) was from Selleckchem; bisbenzimide H 33,342 (Hoechst, B2261), thymidine (T9250), fibronectin (F1141-5 mg) and CK666 (SML0006) were from Sigma-Aldrich.

### 4.4. Western Blot

Immunoblotting was performed as described before [54]. Briefly, cells were lysed in SDS sample buffer (62.5 mM Tris-HCl, pH 6.8, 2.3% SDS (wt/vol), 10% glycerol (vol/vol), 5% β-mercaptoethanol (vol/vol), 0.05% bromphenol blue (wt/vol), 1 mM phenylmethylsulfonylfluoride). Protein concentrations were determined using the DC (detergent compatible) protein assay (Bio-Rad Laboratories, Hercules, CA, USA). Equal amounts of protein from whole cell lysates were separated by SDS-PAGE and electrotransferred onto nitrocellulose membranes. Membranes were blocked with 5% (wt/vol) milk (Nestlé) in Tris-buffered saline (TBS)-polyoxyethylene sorbitan monolaurate (Tween; JT Baker). Membranes were then incubated overnight at appropriate dilution in TBS-Tween/5% milk with the primary antibodies, followed by the horseradish peroxidase (HRP)-conjugated secondary antibody for 1 h at room temperature. Images of the blots were acquired on a MultiImager using the QuantityOne software version 4.5.0 (Bio-Rad Laboratories, Hercules, CA, USA).

### 4.5. Immunofluorescence, PLA, Time Lapse Imaging and Images Quantification

Cells were fixed with 4% (wt/vol) paraformaldehyde (PFA) in PBS for 20 min at room temperature. DNA was stained with cell permeable Hoechst (150 ng/mL). Specimens were processed for immunofluorescence as described [55] and were post-fixed with 3.7% formaldehyde in PBS for 20 min at room temperature. The epifluorescence images were acquired on a AxioObserver Z1 system using a 60x 1.25NA objective or a 40x Plan-Neofluoar 0.6 NA objective and a charged coupled device (CCD) camera Axiocam MRm controlled by the Zen software (ZEISS Group, Baden-Wurttenberg, Germany). Phenotypes were routinely monitored by visual inspection of fixed specimens by at least two independent investigators. Mitotic rounding defects were identified as metaphasic cells presenting a partially round or flat morphology.

For PLA experiments, samples were processed using the Duolink in situ kit (DUO92002-100RXN, Invitrogen, Thermo Fisher Scientific, MA, USA) according to the manufacturer’s instructions in 1% milk PBS, using anti-cortactin (ab33333, abcam) and anti-GFP (632593, Clontech). Confocal microscopy was performed with a PerkinElmer Life Science Ultraview Spinning Disk Confocal microscope, equipped with a 60× oil 1.4NA objective, an Electron Multiplying cooled charge-coupled (EMCCD) camera at −50 °C (Hamamatsu Photonics K.K) and driven by Volocity software (version 6.01; Quorum Technologies, Nikon Group, Tokyo, Japan). Quantification of the PLA foci was performed using Volocity software. First, an average intensity threshold was applied on the GFP-HDAC6 channel to discriminate cells to be analyzed. Only cells between 32 and 250 fluorescence units were retained to avoid analyzing overexpressing cells. Then, the PLA fluorescence channel was binarized using an automatic threshold. The binarized image was processed using Volocity “Find object” tool. To exclude random noise from the detection, a minimum object volume threshold of 0.1 µm^3^ (corresponding to 4 × 4 × 4 pixels) was used.

For live cell imaging, confocal microscopy was performed on the Ultraview Spinning Disk Confocal microscope using a 40x 0.75NA objective equipped with an environmental chamber (humidified/5% CO2/thermoregulated; Nikon Group, Tokyo, Japan). Images were acquired during 75 min at 90 sec-intervals. Subcortical actin cloud phenotypes were determined by visual inspection as follows: sustained and unidirectional motion = unidirectional; changes in the direction of the rotational motion during mitosis = random; and immobile or absent = absent. The defective mitosis phenotype category was defined as the cells presenting mitotic spindle rocking and/or delay in the progression of prometa- and metaphase over the course of the time lapse video.

To quantify the actin cloud angular width, image analysis was performed with FIJI (Fiji Is Just ImageJ, open source image processing package). A point in the center of each mitotic cell was selected with the multipoint tool. A line (length of 25 µm and 1 pixel wide) drawn from this center point was then automatically scanned in 1 degree-steps in a clockwise manner to generate an image in polar coordinate of the actin profile (Figure S2A). A line was then drawn on the resulting actin image in radial coordinate. The thickness of the line was adjusted for each cell depending on the actin cloud penetrance within the cell and the actin angular signal was extracted with the plot profile tool. Finally, a Gaussian regression fit was used to estimate the width of the actin signal variation within the cell, corresponding to the actin cloud angular width (Figure S2B).

### 4.6. Statistical Analysis

Statistical analysis of binomial and multinomial datasets was achieved using the Fisher exact test or chi-square test and the Bonferroni post-hoc analysis to correct for multiple comparisons. Statistical analysis of numerical data was performed using the Mann–Whitney test or the Kruskal–Wallis test followed by the Dunn’s multiple comparisons post-hoc analysis when appropriate. Statistical calculations were performed using Prism 7.0 (GraphPad software, San Diego, CA, USA) statistical software. *p*-values < 0.05 were considered significant (*, *p* < 0.05; **, *p* < 0.01; ***, *p* < 0.001; ****, *p* < 0.0001).

## 5. Conclusions

In conclusion, this study provides novel insights into how BAG3 and its mitotic partners contribute to the fidelity of actin remodeling using mitosis as a paradigm, by uncovering a novel role in tuning the mitotic activity of HDAC6 and cortactin to limit branched actin network assembly. Furthermore, it identifies HDAC6 and cortactin as regulators of the mitotic actin cloud. Future work will be aimed at clarifying the relationship with BAG3-regulated PQC and sequestering activity. It will be interesting to explore the relevance of these findings in other processes regulated by BAG3 and its PQC partners, such as metastatic cell migration.

## Data Availability

All data that support the findings of this study are available from the corresponding author (josee.lavoie@crchudequebec.ulaval.ca) upon reasonable request.

## Supplementary Materials

The following are available at https://www.mdpi.com/1422-0067/22/1/142/s1 Figure S1: BAG3, HSPB8, p62/SQSTM1 or HDAC6 silencing interferes with mitotic cell rounding. (A) Representative Western blots showing the depletion of BAG3, HSPB8 and p62/SQSTM1 at 24 h or 48 h after siRNAs transfection. Depletion was estimated at > 75% based on the linear response from control extract dilutions (siCtl). Vinculin and GAPDH were used as loading controls. (B) Graph showing the percentage of mitotic cells with rounding defects (flat or partially rounded cells) after 48 h depletion of BAG3 or 24 h depletion of p62/SQSTM1 or HSPB8. Data are means ± SEM (*n* = 609 to 817 cells from 3 independent experiments). Statistical analysis was performed by Fisher exact test; *, *p* < 0.05 and ****, *p* < 0.0001. (C) Representative Western blots showing the depletion of HDAC6 along with the corresponding percentage of mitotic cells with rounding defects (flat or partially rounded cells, *n* = 110 to 160 cells from a representative experiment) after 48 h depletion. The depletion in the Western blot was estimated at >75% based on the linear response from control extract dilutions (siCtl); GAPDH was used as loading control. D) Representative Western blots showing the impact of tubacin treatment (5 µm, 8 h) on tubulin acetylation levels as a readout of tubacin efficiency in synchronized HeLa cells treated with the indicated siRNAs and processed for mitotic cell rounding assays, Figure S2: Procedure for the quantification of the actin cloud width. (A) Step 1—actin radial scan: a line drawn (in yellow, siCtl left panel) from the center of the cell is scanned in 1° increments in a clockwise manner to generate an image of the actin in polar coordinate. The right panels show the original image while the left panels show the corresponding polar projection of the cells. The actin cloud is identified in each picture. (B) Step 2—actin cloud angular width estimation: a line was drawn (from 1 to 2 or from 2 to 1 depending the cell) on the polar coordinate images along the angle direction and the actin intensity profile was extracted along this line. Finally, a Gaussian fit was used to estimate the actin cloud angular width parameter from the actin intensity profile of the cell, Figure S3: Arp2/3 inhibition by CK666 treatment normalizes mitotic cell rounding in BAG3 depleted cells. (A) Schematic of the protocol to perform the mitotic cell rounding knockdown-rescue experiments. (B) Graph depicting the percentages of mitotic cells with rounding defects (flat or partially rounded cells); means ± SEM (*n* = 200 to 214 cells from a representative experiment), statistical analysis was performed by the chi-square test; ****, *p* < 0.0001. Figure S4: (A) A high dose of tubacin prevents the formation of the actin cloud. Graph left panel: percentage of cells showing a normal or defective motion of the subcortical actin cloud or a static or lacking subcortical actin cloud (absent). Graph right panel: percentage of cells with normal or defective mitosis (spindle rocking and/or delay in prometa- or metaphase) along with their corresponding proportions of subcortical actin cloud movement phenotypes; data are means ± SEM (*n* = 46 and 72 cells from a representative experiment), statistical analysis was performed by the chi-square test; **, *p* < 0.01; ****, *p* < 0.0001. (B) Acetyl-mimic cortactin-9KQ can normalize mitotic cell rounding in HSPB8-depleted cells. Representative deconvolved epifluorescence images showing the mitotic rounding phenotypes induced by cortactin mutants. The graph depicts the percentages of cells with defects in mitotic cell rounding; means ± SEM of at least 609 cells from 3 experiments, same siCtl as in Figure 5B. Statistical analysis was performed by the chi-square test ****, *p* < 0.0001, Video 1: Unidirectional motion of the subcortical actin cloud in siCtl cells expressing LifeAtc-GFP, Video 2: Random motion of the subcortical actin cloud, stained by LifeAct-GFP, typically observed in cells depleted for BAG3, HSPB8 or p62/SQSTM1.

## Abbreviations

PQC	protein quality control
PLA	proximity ligation assay
TLA	three letter acronym
LD	linear dichroism
GFP	Green Fluorescent protein
RFP	Red Fluorescent Protein
